# Dr. Joseph N. Strange

**Published:** 1912-10

**Authors:** 


### OBITUARIES.

Dr. Joseph N. Strange (licence, Arkansas, 1903 of Hartford, was shot and killed in a pistol duel in Hartford, Aug. 24, aged 45 *Jour. A. M. A.*) And this is 1912.

